# Molecular‐Informed Network Analysis Unveils Fatigue‐Related Functional Connectivity in Parkinson's Disease

**DOI:** 10.1002/mds.30214

**Published:** 2025-04-22

**Authors:** Ilaria Antonella Di Vico, Manuela Moretto, Agnese Tamanti, Giovanni Tomelleri, Giulia Burati, Daniel Martins, Ottavia Dipasquale, Mattia Veronese, Alessandra Bertoldo, Elisa Menini, Angela Sandri, Sarah Ottaviani, Francesca Benedetta Pizzini, Michele Tinazzi, Marco Castellaro

**Affiliations:** ^1^ Neurology Unit, Department of Neurosciences, Biomedicine and Movement Sciences, Policlinico Borgo Roma University of Verona Verona Italy; ^2^ Department of Information Engineering University of Padova Padova Italy; ^3^ Institute of Psychiatry, Psychology & Neuroscience, Department of Neuroimaging, King's College London Institute of Psychiatry London UK; ^4^ Olea Medical La Ciotat France; ^5^ Padova Neuroscience Center University of Padova Padova Italy; ^6^ Neurology Unit, Policlinico Borgo Trento Verona Verona Italy; ^7^ Department of Engineering for Innovation Medicine University of Verona Verona Italy

**Keywords:** functional connectivity, Parkinson's disease, neuroreceptor system, resting state fatigue

## Abstract

**Background:**

Fatigue in Parkinson's disease (PD) is a prevalent and debilitating non‐motor symptom. Despite its significant impact on quality of life, the underlying neurochemical and network‐based mechanisms remain poorly understood.

**Objectives:**

This observational study applied a multimodal imaging approach to explore potential links between the functional connectivity of neurotransmitter‐specific circuits and fatigue in a sample of patients with PD.

**Methods:**

We acquired resting‐state functional magnetic resonance imaging data in 35 patients with PD including 18 with clinically significant fatigue and 17 without. We applied the receptor‐enriched analysis of functional connectivity by targets (REACT) pipeline to derive patients' specific molecularly enriched networks informed by the spatial distribution of the dopamine, noradrenaline, serotonin transporters, and metabotropic glutamate 5 receptors as assessed using molecular imaging data in independent samples of healthy controls. We then conducted whole‐brain analyses inspecting both categorical differences between groups of patients with and without clinically significant fatigue, and associations exploring the full within‐sample variation in symptom ratings.

**Results:**

We found a significant decrease in noradrenaline‐enriched and glutamate‐enriched functional connectivity in key regions, belonging to the sensorimotor, salience, and default mode network, with increasing fatigue severity. Notably, noradrenaline‐enriched functional connectivity reductions were widespread, while glutamate‐enriched functional connectivity reductions were more restricted to the supplementary motor area. No significant relationships between fatigue and dopamine or serotonin‐enriched functional connectivity were found.

**Conclusions:**

These findings offer supportive evidence for the putative involvement of the noradrenaline and glutamate systems in the genesis of fatigue in PD, opening new directions for treatment development exploring these neurochemical systems. © 2025 The Author(s). *Movement Disorders* published by Wiley Periodicals LLC on behalf of International Parkinson and Movement Disorder Society.

Fatigue in Parkinson's disease (PD) is a common, pervasive, and debilitating symptom, yet its pathophysiology remains poorly understood.^1^, ^2^ Defined as an overwhelming lack of energy or a need for increased effort during daily activities, fatigue significantly impacts the quality of life of almost half of individuals living with PD.^1^ Despite its clinical significance, current efforts to identify effective treatments have been hampered by our limited understanding of the underlying neurobiology, where the subjective nature of this symptom and the intrinsic overlap with other symptoms, such as apathy or depression, pose significant challenges limiting scientific advance.^3^, ^4^

The application of neuroimaging techniques, such as resting‐state functional magnetic resonance imaging (rs‐fMRI), offers valuable insights into the neural correlates of fatigue. For instance, studies have revealed alterations in the functional connectivity (FC) of the default mode (DMN) and somatomotor networks (SMN) between patients with and without fatigue, suggesting that dysfunction within these circuits may contribute to both motor and cognitive impairments associated with fatigue in PD.^5^ Notably, areas involved in motor planning and preparation, including pre‐motor cortices, the supplementary motor area (SMA), and prefrontal and posterior cingulate areas have been implicated in distressing fatigue in drug‐naïve PD patients.

Building on these findings, Siciliano and colleagues^6^ employed rs‐fMRI to investigate the brain FC of SMA subregions in de novo, drug‐naïve PD patients with fatigue. Their study revealed increased connectivity between the left pre‐SMA and the left postcentral gyrus in patients with fatigue compared with patients without fatigue, suggesting abnormal sensory processing during voluntary movements.^6^ Additionally, decreased connectivity between the left SMA proper and the left middle frontal gyrus highlighted the role of attentional network dysfunction in fatigue pathology, confirmed by neurophysiological studies demonstrating difficulty in attentional orienting to salient novel stimuli in PD‐fatigued patients.^7^ These findings align with a pathological model of fatigue suggesting that poor attenuation of sensory signals from somatosensory systems to higher‐order motor systems may contribute to an amplified sense of effort.^8^, ^9^, ^10^

Moreover, recent conceptualizations propose that fatigue in neurological disorders, including PD, may be related to altered central processing of sensory input, specifically altered sensory attenuation.^10^, ^11^ Perceived effort, a measure of the brain's estimated action cost, is elevated in PD and other neurological diseases, contributing to fatigue despite similar muscle output or energy expenditure.^11^

Drawing parallels with findings in other neurological diseases, such as multiple sclerosis (MS), helps elucidate the complex interplay between neurotransmitter systems and fatigue. For instance, decreased noradrenaline transporter (NET)‐enriched connectivity in frontal regions of MS patients with cognitive fatigue implicates noradrenergic dysfunction in fatigue pathogenesis.^12^ The locus coeruleus, the primary source of noradrenaline in the brain, is critical for arousal, attention, and cognitive functions. While its direct involvement in PD‐related fatigue remains inconclusive,^13^, ^14^ its widespread projections and role in regulating motivation and working memory suggest a potential contribution to fatigue pathogenesis and treatment response.^12^, ^13^, ^14^

Beyond the noradrenergic system, other neurotransmitters have been implicated in PD‐related fatigue, including glutamate, serotonin, and dopamine.^15^, ^16^, ^17^ Glutamatergic hyperactivity contributes to neuroinflammation, neurodegeneration,^16^, ^17^ and non‐motor symptoms.^18^ Safinamide, a monoamine oxidase‐B inhibitor with additional glutamate‐modulating properties, has shown promise in alleviating fatigue, possibly through its dual action on dopaminergic and glutamatergic pathways.^19^, ^20^ While serotonergic alterations have been observed in fatigued PD patients with positron emission tomography (PET) studies showing reduced serotonin transporter (SERT) binding in basal ganglia and limbic regions,^15^ selective serotonin reuptake inhibitors have shown limited efficacy in fatigue management.^21^ This suggests that serotonergic dysfunction alone may not fully explain its pathophysiology. Dopaminergic dysfunction appears to play a minor role, as indicated by the poor correlation between fatigue severity, levodopa equivalent daily dosage (LEDD), and dopamine transporter (DAT) availability in L‐dopa‐naïve PD patients.^1^, ^15^

Despite profound advancements in the comprehension of fatigue, there is a big gap in understanding pathophysiological and molecular mechanisms of fatigue in PD, especially concerning patients in non‐early stages of the disease and those under stable dopaminergic therapy. Moreover, there is a lack of consistently replicated data on the most effective medications to treat fatigue in PD, limiting available therapeutic options.^21^, ^22^

To address these gaps, we have adopted the innovative approach of receptor‐enriched analysis of functional connectivity by targets (REACT)^23^ to integrate molecular neuroimaging data into our rs‐fMRI analysis and therefore enhance the interpretation of functional connectivity by incorporating high‐resolution PET templates that map the spatial distribution of specific neuroreceptors across the brain.

This method allowed us to assess how brain networks are ‘enriched’ for specific receptor or transporter distributions, providing a nuanced delineation of the functional networks modulated by distinct neurotransmitter systems, thereby enabling deeper insights into the molecular substrates underpinning observed connectivity patterns.

Originally developed to study pharmacological activation via receptor distributions,^23^ the REACT methodology has already been applied to investigate molecular FC in MS‐related fatigue.^12^ Here, we extend this approach to PD, leveraging rs‐fMRI and molecular templates to examine neurotransmitter‐specific circuits.

The aim of the present study was to investigate how neural networks related to the main brain neurotransmitter transporters (DAT, NET, SERT) and metabotropic glutamate receptor 5 (mGluR5), affect the perception of fatigue in a cohort of 35 PD patients, including 18 with clinically significant fatigue (FAT) and 17 without (NFAT). Gaining a better understanding of these relationships may lead to new insights into the underlying mechanisms of fatigue in PD and potentially identify new targeted therapeutic strategies.

## 1 Methods

### 1.1 Participants

The study involved 35 consecutive patients diagnosed with idiopathic PD who were enrolled at the Neurology Unit, Movement Disorders Division, University of Verona, Italy. Recruitment was consecutive but temporarily interrupted during COVID‐19 lockdowns, resulting in brief gaps in enrollment. Participants were aged between 36 and 86 years and had mild‐to‐moderate PD, as determined by the Modified Hoehn & Yahr scale (Stages 1–3).^24^ Exclusion criteria were the presence of pacemakers or ferromagnetic implants, a Montreal Cognitive Assessment (MoCA)^25^ score below 21 (indicating dementia), or a history of severe psychiatric disorders. Written informed consent was obtained from all participants, and the research protocol was approved by the ethical committee of the University Hospital of Verona (Project No. 2899, Approval No. 48632, 14/09/2020), adhering to the principles of the Helsinki Declaration.

### 1.2 Clinical Assessment

Demographic information, including age and gender, was collected during clinical assessments, along with disease duration and LEDD. Fatigue severity was assessed using the Fatigue Severity Scale (FSS), a nine‐item, easy‐to‐administer scale that is the only one recommended for screening and grading fatigue severity in PD and in various neurological disorders.^26^, ^27^ A cut‐off of FSS > 4 was used to classify patients as experiencing clinically significant fatigue.

Additionally, scales for other non‐motor symptoms were administered to control for potential confounding factors. In particular, depression and anxiety were assessed using the Hospital Anxiety (HAS) and Depression Scale (HDS),^28^ with a cut‐off score ≥ 11 indicating probable impairment in either domain.^29^, ^30^ Apathy was measured using the Apathy Evaluation Scale (AES),^31^, ^32^ where a score ≥ 40 was considered indicative of clinically significant apathy. Excessive daytime sleepiness was assessed with the Epworth Sleepiness Scale (ESS), and a cut‐off score of ≥10 was used to define excessive daytime somnolence.^33^ Finally, sleep disturbances were evaluated using the Parkinson's Disease Sleep Scale (PDSS‐2), with a threshold of ≥18 indicating poor sleep quality.^33^

Disease severity was evaluated using the Movement Disorder Society‐sponsored revision of the Unified Parkinson's Disease Rating Scale‐Part III (UPDRS‐III).^34^ All assessments were conducted while patients were receiving their regular dopaminergic treatment. The total dosage of these medications was standardized by converting it to the LEDD.^35^

### 1.3 MRI Data Acquisition

Images were acquired at the University Hospital of Verona, on a 3 T Philips Elition S scanner equipped with a 32‐channel head coil. The magnetic resonance brain imaging protocol included: a structural T1‐weighted (T1w) Turbo Field Echo sequence (TR/TE 8.2/3.7 ms, 1 × 1 × 1 mm^3^; Compressed SENSE = 3); approximately 10 min of rs‐fMRI acquired with a single‐shot Echo‐Planar Imaging sequence (TR/TE 1100/30 ms, FA = 64°, 2.4 × 2.4 × 2.4 mm^3^, MultiBand SENSE = 4, SENSE = 2, anterior–posterior phase encoding direction, 500 volumes); two spin echo Echo‐Planar Imaging sequences acquired with inverted polarity of the phase encoding direction for distortion correction purposes and geometrically matched with the rs‐fMRI sequence (SENSE = 2, anterior–posterior and posterior–anterior phase encoding directions).

### 1.4 MRI Data Preprocessing

Both anatomical and functional data were preprocessed with in‐house pipelines.

The anatomical pipeline applied to the T1w structural image included N4 bias field correction,^36^ skull‐stripping (MASS^37^) and nonlinear diffeomorphic registration^38^ to the nonlinear MNI152 2006 atlas. From the preprocessed structural data, we then obtained the estimated intracranial volume (eICV) using Freesurfer (v7.1.0), run with default parameters.^39^

The functional pipeline included: (1) slice timing correction^40^; distortion correction (TOPUP)^41^; motion correction (MCFLIRT)^42^; regression for nuisance variables (six head motion parameters, average cerebrospinal and white matter signals); despiking to correct spikes representing artifacts induced by motion (considering a framewise displacement threshold of 0.4 mm), and temporal high‐pass filtering (cut‐off frequency = 1/128 Hz). Finally, data were smoothed with a 5 mm^3^ Gaussian kernel and normalized into the nonlinear MNI152 2006 standard space at 2 × 2 × 2 mm^3^ resolution.

Before proceeding with the subsequent analyses, all the preprocessed rs‐fMRI data underwent a visual quality check. Head motion, quantified by mean framewise displacement, was found to be below 0.3 mm in all subjects. As a result, all data were retained for further analyses.

### 1.5 Neuroreceptor‐Enriched Functional Connectivity

For the REACT analysis,^23^ we used publicly available molecular templates of the DAT,^43^ NET,^44^ SERT,^45^ and mGluR5^46^ molecular systems. The DAT template was derived from [^123^I]‐ioflupane single‐photon emission computerized tomography images from 30 healthy subjects without evidence of nigrostriatal degeneration. The NET template was obtained by averaging the [^11^C]‐MRB PET brain parametric maps from a cohort of 10 healthy subjects. The SERT template was derived from [^11^C]‐DASB PET images from 210 healthy subjects and, finally, the mGluR5 template from [^11^C]‐ABP688 PET parametric maps from a cohort of 31 healthy subjects. All the templates were normalized by scaling the map values between 0 and 1 and masked using the FSL standard grey matter mask with a threshold of 0.3. Then, the reference regions used for quantification of the molecular data in the kinetic model, namely the occipital areas for DAT and NET and the cerebellum for SERT, were masked out.

To investigate changes in FC related to each molecular system, the REACT pipeline employs a two‐step multiple regression analysis.^47^

Specifically, in the first step, the four molecular templates are used as a set of spatial regressors to weigh the subject‐specific rs‐fMRI volumes and estimate the dominant BOLD fluctuation related to each molecular system. In the second step, the subject‐specific time series obtained previously are used as temporal regressors to estimate the subject‐specific spatial maps associated with each molecular system. Thus, after running REACT, four spatial maps reflecting the subject‐specific molecular‐enriched FC are obtained.

### 1.6 Statistical Analysis

#### 1.6.1 Demographics, Clinical, and Experimental Variables

Normality of clinical data was assessed using the Shapiro–Wilk test, setting a significance level to *P* < 0.05 (two‐tailed) and then Pearson's correlation between clinical scales was computed.

We compared variables between the two patient groups (FAT: fatigued patients with FSS score > 4; NFAT: non‐fatigued patients with FSS score < 4) using *χ* ^2^ tests for gender; independent samples *t*‐tests for age, FSS, PDSS‐2, ESS, HDS, MoCA scores and eICV; and Mann–Whitney tests for nonparametric variables such as disease duration, LEDD, HAS, AES, and MDS‐UPDRS‐III.

#### 1.6.2 *T*‐Test on Molecular‐Enriched FC Maps between FAT and NFAT Patients

Then, we conducted a two‐sample unpaired voxel‐wise *t*‐test at the whole‐brain level to examine differences in molecular‐enriched FC between patients with (FAT) and without (NFAT) clinically significant fatigue. Age, gender, LEDD, and eICV were included as nuisance variables.

#### 1.6.3 Within‐Group Associations between Molecular‐Enriched FC and FSS

Next, we tested the association between variations in molecular‐enriched FC and FSS, using two different regression models, which differed in the nuisance variables included. In the first model, we included only age, gender, LEDD, and eICV, while in the second model we added AES scores as an additional nuisance variable.

For DAT‐enriched FC maps only, these two models were also repeated after excluding LEDD from the nuisance variables to eliminate potential bias in dopamine‐related results driven by treatment.

#### 1.6.4 Within‐Group Associations between Molecular‐Enriched FC and HDS/HAS

Finally, employing the same regression methods, we tested the association between variations in molecular‐enriched FC and depression (HDS) or anxiety (HAS) scores, using HDS or HAS as explanatory variables, controlling for age, gender, LEDD, and eICV.

Statistically significant voxel clusters were labeled by computing the normalized overlap between these clusters and (a) networks defined in Schaefer's functional atlas^48^ and (b) regions defined in the AAL3 anatomical atlas.^49^

Finally, for significant associations, we extracted the mean FC value from clusters that were significantly associated with FSS to estimate Pearson's correlation coefficient. These values are reported as effect size measures rather than for statistical inference, as such an analysis would be circular.

For all the analyses described in points 1.6.2, 1.6.3, and 1.6.4 we used nonparametric, permutation‐based statistical tests implemented in FSL's PALM.^50^ PALM makes minimal assumptions about data distribution, ensuring robust and reliable statistical inference when dealing with complex neuroimaging data. To expedite the permutation test, we limited the number of permutations to 500 and employed the tail approximation approach.^51^ We applied threshold‐free cluster enhancement (TFCE)^52^ to detect statistically significant clusters (*P* < 0.05) of voxels, ensuring familywise‐error control. The TFCE parameters were set to H = 2, E = 2, C = 6.

## 2 Results

### 2.1 Demographics, Clinical, and Experimental Variables

We found no significant differences between FAT and NFAT patients for gender, disease duration, LEDD, PDSS‐2, ESS, MDS‐UPDRS‐III, and eICV. There was a significant difference between FAT and NFAT patients in age, FSS, HAS, HDS, and in the AES (Table 1). Significant Pearson's correlations were found between FSS and both HDS and HAS; HDS and HAS and AES and MoCA scores; and HAS and AES (Table 2). It is worth noting that, in our cohort, only one patient had an HDS score above the cut‐off value of 11, while four patients had HAS scores exceeding the same threshold.

**TABLE 1:** Demographic and clinical information of the study sample

Variable	Group			Statistics	
	Total sample Mean ± SD [min, max]	NFAT Mean ± SD [min, max]	FAT Mean ± SD [min, max]	*t*	*P*‐value
N	35	17	18		
Age (years)	64.63 ± 9.99 [36, 83]	61.06 ± 10.88 [36, 77]	68 ± 7.98 [55, 83]	−2.16	0.038
Gender (F/M)	17/18	7/10	10/8	*χ* ^2^ = 1.52	0.218
Disease duration (years)	4.73 ± 3.36 [0.5, 15]	4.53 ± 1.18 [1, 8]	4.92 ± 4.25 [0.5, 15]	Mann–Whitney U = 167.6	0.64
FSS	4.08 ± 1.37 [1.1, 6.56]	2.92 ± 0.75 [1.11, 3.89]	5.18 ± 0.79 [4, 6.56]	−8.66	<0.001
LEDD	483.77 ± 342.56 [50, 1460]	507.41 ± 375.18 [52, 1350]	461.44 ± 318.01 [50, 1460]	Mann–Whitney U = 156.5	0.92
PDSS‐2	11.25 ± 5.70 [0, 24]	10.92 ± 5.95 [0, 22]	11.56 ± 5.62 [3, 24]	−0.33	0.747
ESS	6.04 ± 2.97 [1, 11]	5.57 ± 3.14 [1, 11]	6.48 ± 2.81 [1, 11]	−0.9	0.374
HAS	5.53 ± 3.70 [1, 13]	3.82 ± 2.63 [1, 11]	7.14 ± 3.91 [1, 13]	Mann–Whitney *U* = 75	0.01
HDS	5.59 ± 2.78 [0, 12]	4.13 ± 1.99 [0, 7]	6.97 ± 2.76 [3, 12]	−3.46	0.001
AES	59.20 ± 9.40 [34, 72]	62.16 ± 9.63 [34, 72]	56.40 ± 8.52 [38, 68]	Mann–Whitney *U* = 219	0.03
MDS UPDRS‐III	15.71 ± 7.52 [3, 34]	13.82 ± 71.6 [3, 27]	17.50 ± 7.60 [8, 34]	Mann–Whitney *U* = 112.5	0.19
MoCA	26.91 ± 2.63 [21, 30]	27.62 ± 2.58 [22, 30]	26.28 ± 2.58 [21, 30]	−1.51	0.14
eICV	1.46 × 10^6^ ± 2.43 × 10^5^ [8.55 × 10^5^, 2.11 × 10^6^]	1.45 × 10^6^ ± 2.29 × 10^5^ [8.55 × 10^5^, 1.75 × 10^6^]	1.46 × 10^6^ ± 2.63 × 10^5^ [1.06 × 10^6^, 2.11 × 10^6^]	−0.11	0.92

Abbreviations: SD, standard deviation; NFAT, non‐fatigued PD patients (ie, with FSS < 4); FAT, fatigued PD patients (ie, with FSS > 4); F, female; M, male; FSS, Fatigue Severity Scale; LEDD, levodopa equivalent daily dose; PDSS‐2, Parkinson's Disease Sleep Scale; ESS, Epworth Sleepiness Scale; HAS, Hospital Anxiety Scale; HDS, Hospital Depression Scale; AES, Apathy Evaluation Scale; MDS‐UPDRS‐III, Movement Disorder Society‐sponsored revision of the Unified Parkinson's Disease Rating Scale‐Part III; MoCA, Montreal Cognitive Assessment; eICV, estimated intracranial volume.

**TABLE 2:** Correlations between clinical scores

Pearson's correlation between clinical scores								
	FSS	PDSS‐2	ESS	HAS	HDS	AES	MDS UPDRS‐III	MoCA
FSS	–							
PDSS‐2	0.059	–						
ESS	0.275	0.106	–					
HAS	0.563***	0.237	0.142	–				
HDS	0.603***	0.232	0.167	0.585***	–			
AES	−0.328	0.144	−0.027	−0.419*	−0.523**	–		
MDS UPDRS‐III	0.21	0.126	−0.21	−0.077	0.246	−0.235	–	
MoCA	−0.24	0.25	0.11	0.05	−0.42*	0.34*	−0.15	–

*Note*: The table reports Pearson's correlation between clinical scores.

Abbreviations: FSS, Fatigue Severity Scale; PDSS‐2, Parkinson's Disease Sleep Scale; ESS, Epworth Sleepiness Scale; HAS, Hospital Anxiety Scale; HDS, Hospital Depression Scale; AES, Apathy Evaluation Scale; MDS‐UPDRS‐III, Movement Disorder Society‐sponsored revision of the Unified Parkinson's Disease Rating Scale‐Part III; MoCA, Montreal Cognitive Assessment.

* *P* <0.05;

** *P* < 0.01;

*** *P* < 0.001.

### 2.2 *T*‐Test on Molecular‐Enriched FC Maps between FAT and NFAT Patients

We did not find any significant differences between FAT (N = 18) and NFAT (N = 17) patients in all the evaluated REACT maps (DAT, NET, SERT, mGluR5). We repeated the test removing the patient with an outlier age in the NFAT group, so that the two groups of patients were no longer significantly different in terms of age. This allowed us to confirm that age differences would not impact the significance of the FAT versus NFAT comparison. The results remained non‐significant (*P* > 0.05).

### 2.3 Within‐Group Associations between Molecular‐Enriched FC and FSS

We observed negative linear relationships between FSS scores and both FC maps enriched by NET and mGluR5, after accounting for age, gender, LEDD, and eICV.

**FIG. 1:**
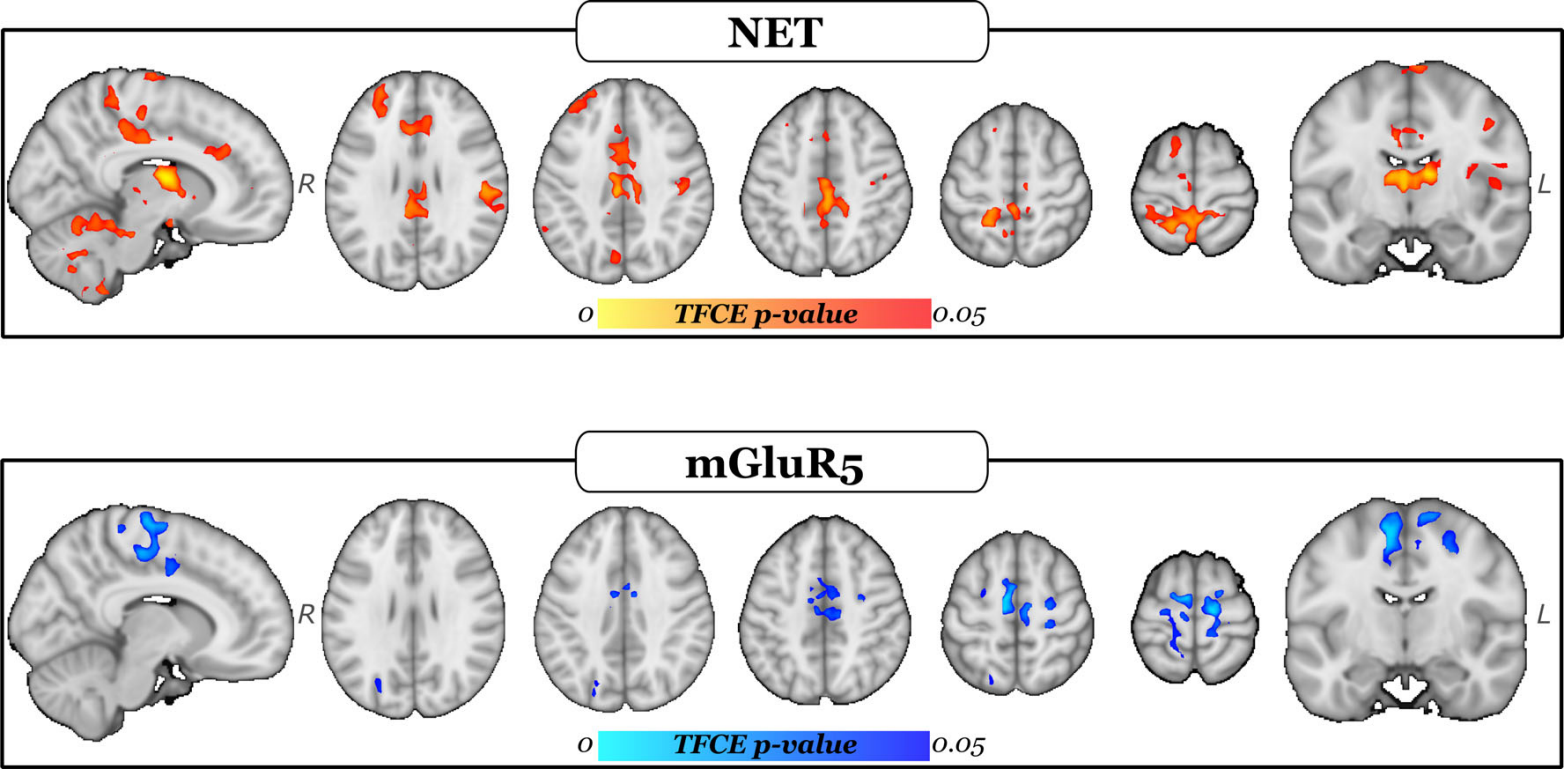
Decreased noradrenaline transporter (NET)‐enriched and metabotropic glutamate receptor 5 (mGluR5)‐enriched functional connectivity (FC) with increasing fatigue. Clusters of statistically significant decrease of NET‐enriched FC (top row) and mGluR5 (bottom row) with increasing fatigue. The color scales represent *P*‐values after correction for multiple comparisons. In the top row, warmer colors indicate more significant voxels. Similarly, in the bottom row, lighter colors indicate more significant voxels. The statistical maps are overlaid onto the MNI atlas. [Color figure can be viewed at wileyonlinelibrary.com]

For NET, we found a significant negative relationship between fatigue and NET‐enriched FC (T_TFCE_ = 831.62, *P* = 0.009), which was widespread in the brain (Fig. 1, top panel). The five regions of the AAL atlas^49^ with the highest overlap with significant clusters were the left and right middle cingulate cortex/gyrus, right cerebellum, left superior frontal cortex/gyrus, and left precuneus (see Supplementary Materials for the complete list).

When assessing the overlay with cortical functional networks, as defined in the Schaefer functional atlas,^48^ we found a major involvement of areas belonging to the SMN (29.6% overlay), salience (28% overlay), and DMN (21.2% overlay).

For mGluR5, we also found a statistically significant negative relationship between fatigue and mGluR5‐enriched FC (T_TFCE_ = 776.59, *P* = 0.026), that is, mGLuR5‐enriched FC decreases with increasing FSS score (Fig. 1, bottom panel). Compared with the NET‐enriched FC maps, for mGluR5‐enriched FC maps, the five regions with the highest overlap were the left and right SMA, right precentral cortex, right middle cingulate cortex, right superior frontal cortex, left middle cingulate cortex, and the left paracentral lobule. The complete list is reported in the Supplementary Materials. Specifically, the cortical functional networks mainly involved were the SMN (60.8% overlay) followed by the salience (23.5% overlay).

After incorporating AES scores into the nuisance variables, significant clusters for NET and mGluR5 were found in the same brain regions as in the models that did not include AES, as reported in the Supplementary Materials.

**FIG. 2:**
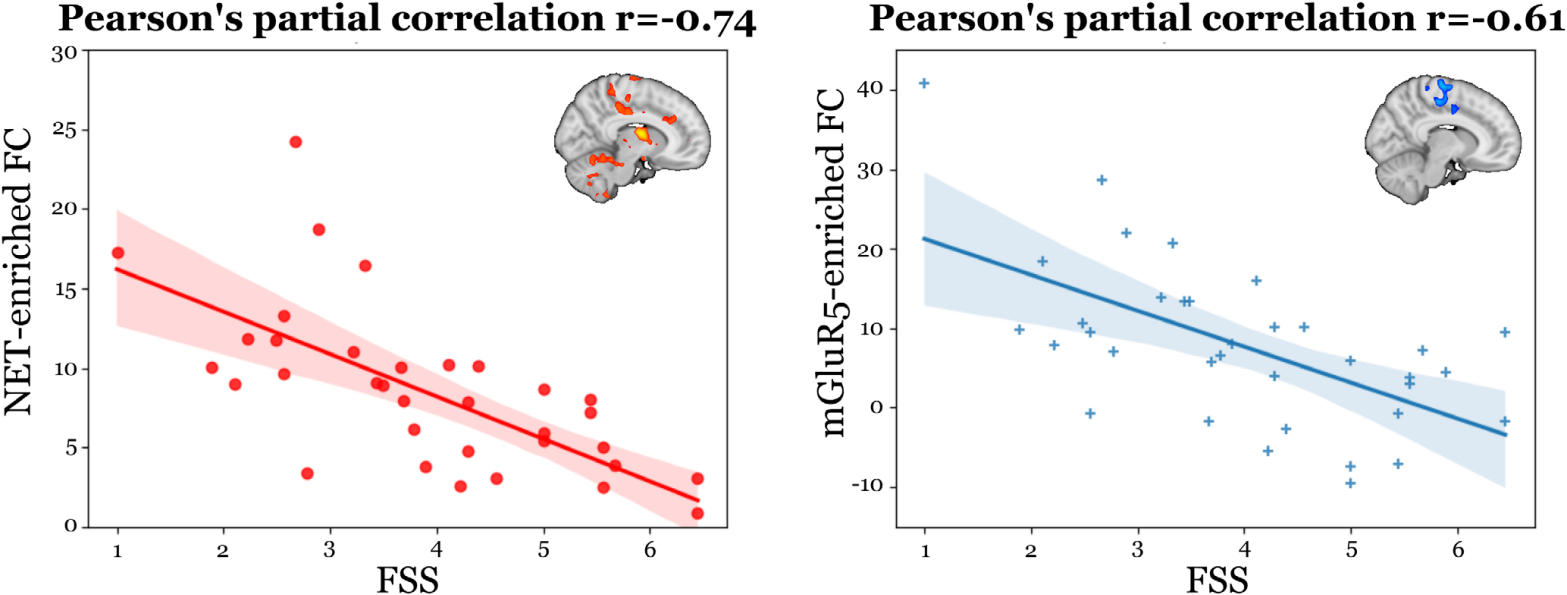
Anticorrelation between noradrenaline transporter (NET)‐enriched and metabotropic glutamate receptor 5 (mGluR5)‐enriched functional connectivity (FC) and interindividual FSS scores. The left panel reports the partial correlation results between Fatigue Severity Scale (FSS) scores and NET‐enriched FC. In the right panel, the partial correlation results between FSS and mGluR5‐enriched FC are shown. [Color figure can be viewed at wileyonlinelibrary.com]

The partial correlation analysis between the average enriched FC extracted within statistically significant voxels resulting in the permutation regression analysis, and FSS scores resulted in a Pearson's correlation of −0.74 for the NET‐enriched FC (Fig. 2, left panel) and of −0.61 for the mGluR5‐enriched FC (Fig. 2, right panel).

We found no significant relationship between FSS and the FC maps enriched for SERT or DAT. For DAT, this result was confirmed even after excluding LEDD from the nuisance variables in the models.

### 2.4 Within‐Group Associations between Molecular‐Enriched FC and HDS/HAS

Given the high correlation between HDS, HAS, and FSS, depression and anxiety scores were excluded from the main models to avoid removing variance shared with fatigue. Instead, sensitivity analyses were performed using HDS or HAS as explanatory variables, controlling for age, gender, LEDD, and eICV.

We found no significant relationship between HAS and FC in any molecular map. However, a significant relationship emerged between HDS and mGluR5‐enriched FC maps (T_TFCE_ = 1015.43, *P* = 0.014), specifically in the left and right SMAs, left precentral and postcentral cortices, left and right paracentral lobules, and the middle cingulate cortex. These regions largely overlapped with areas also correlated with FSS (see Supplementary Materials).

## 3 Discussion

In this study, we employed a multimodal approach to investigate changes in FC associated with neurochemical‐enriched circuits of dopamine, noradrenaline, mGluR5, and serotonin transporter in PD patients with varying levels of fatigue.

Our primary finding revealed a coupled reduction of the connectivity of mGluR5 and NET in key regions such as the SMN, DMN, and Salience network. Specifically, both noradrenaline‐enriched FC and mGluR5‐enriched FC demonstrated a significant decrease as fatigue levels increased, with mGluR5‐FC being particularly localized in the SMA. Moreover, NET‐enriched connectivity exhibited widespread reductions across the SMN, salience, and DMN.

However, since no significant group differences in neurotransmitter‐enriched connectivity were observed, these findings should be interpreted with caution. The observed alterations may reflect broader neurotransmitter dysfunctions rather than mechanisms exclusively driving fatigue. Nevertheless, our results provide novel evidence that higher fatigue levels in PD are associated with reduced connectivity in noradrenergic and glutamatergic circuits, suggesting that these networks may contribute to fatigue perception.

While this study does not establish a direct causal relationship, it highlights potential neurochemical substrates involved in fatigue expression.

Importantly, this modulation persisted even after accounting for potential confounding factors, such as anxiety or apathy, which are known to influence fatigue perception. This reinforces the hypothesis that neurotransmitter‐related circuits, particularly those linked to noradrenaline and glutamate, may play a role in PD fatigue pathophysiology beyond simple mood or motivational disturbances. However, given the lack of direct PET imaging data from PD patients, further studies using patient‐specific molecular imaging are warranted to confirm these findings.

### 3.1 Neurotransmitter‐Specific Findings

Our findings on reduced mGluR5‐enriched connectivity are particularly intriguing when considering the context of excitotoxicity. The receptor mGluR5 is known to modulate excitatory transmission, primarily through interactions with N‐methyl‐D‐aspartate receptors.^53^ Typically, increased mGluR5 activity is linked to heightened excitatory neurotransmission, leading to excitotoxic stress on neurons—a process associated with neurodegeneration in PD.^53^ Therefore, the observed reduction in mGluR5 activity in our study might be seen as a compensatory mechanism to alleviate this excitotoxic burden.

However, despite the reduction in mGluR5‐enriched connectivity, fatigue persisted in these patients, suggesting that simply reducing excitatory drive is insufficient to resolve non‐motor symptoms like fatigue. This implies that fatigue in PD may arise from a more complex interplay between excitatory and inhibitory systems or may involve other glutamatergic receptors (eg, N‐methyl‐D‐aspartate or α‐amino‐3‐hydroxy‐5‐methyl‐4‐isoxazolepropionic acid receptor) that are also crucial for maintaining neural balance.

While reduced mGluR5 transmission may mitigate excitotoxicity, an overall imbalance in glutamatergic signaling likely continues to drive the sensation of fatigue. Moreover, clinical trials targeting mGluR5 with negative allosteric modulators have faced challenges, as these negative allosteric modulators, which reduce excitatory drive in the basal ganglia, have shown limited success and may interfere with L‐dopa efficacy for motor symptoms.^54^ This suggests that the role of mGluR5 and glutamatergic systems in fatigue is likely broader and involves more than just excitotoxicity.

We also observed a widespread reduction in NET‐enriched FC, particularly in areas associated with both motor and cognitive processes, such as the SMN, salience, and DMN. This might be linked to a primary reduction in NET activity within the locus coeruleus, a region with extensive projections throughout the brain that plays a crucial role in regulating high‐order cognitive functions, arousal, and motivation.

While earlier studies^13^ linked locus coeruleus atrophy to depression and vigilance rather than fatigue itself, more recent research using advanced techniques like hybrid MRI/PET has demonstrated a direct correlation between neuromelanin degeneration in the locus coeruleus and fatigue in PD patients.^14^ This suggests that the role of locus coeruleus degeneration in PD‐related fatigue may be more significant than previously believed.

Interestingly, studies on MS^12^ have shown that reduced NET connectivity in frontal regions is closely linked to the onset of cognitive fatigue, highlighting the role of noradrenaline dysfunction in fatigue mechanisms across different neurological diseases. This suggests that the noradrenergic system could have a broader role in fatigue pathophysiology, not just in PD but across various conditions, underscoring the transdiagnostic importance of neurotransmitter imbalances in both motor and non‐motor symptoms.

Future transdiagnostic studies are essential to determine whether this represents a general feature of fatigue across neurological diseases or if there are diagnosis‐specific mechanisms at play, helping to clarify whether fatigue shares common neurochemical underpinnings across conditions.

Our analysis revealed that mGluR5‐enriched FC correlated with both FSS (fatigue) and HDS (depression), whereas NET‐enriched FC showed a stronger and more specific correlation with FSS alone. This suggests that the noradrenergic system may be more specifically implicated in the pathophysiology of fatigue, independent of depressive or anxiety symptoms, while the glutamatergic system may be less specifically involved in both fatigue and affective symptoms, like depression. The fact that only one patient exceeded the clinical threshold for depression and four patients for anxiety further reinforces the idea that NET disruptions are more fatigue‐specific, while mGluR5 dysfunction might reflect a shared neurochemical mechanism underlying both fatigue and mood disturbances.

These findings highlight the potential of noradrenergic circuits as targeted treatment pathways for fatigue, independent of mood‐related treatments, while glutamatergic dysfunction may require more comprehensive therapeutic strategies.

While dopamine dysfunction is often implicated in PD‐related motor symptoms, our results confirm that LEDD and DAT‐enriched FC were not significantly associated with fatigue severity. This aligns with prior studies showing weak correlations between fatigue and dopaminergic deficits, suggesting that dopamine alone is unlikely to be the primary driver of this symptom.^1^

Similarly, although previous research has highlighted serotonergic alterations in PD‐related fatigue,^1^, ^15^ our study found no significant correlation between fatigue and SERT‐enriched FC. This finding challenges the long‐standing serotonergic hypothesis of PD fatigue, suggesting that serotonin dysfunction may not play as dominant a role as previously hypothesized.

Importantly, our findings emphasize the need to move beyond the traditional focus on dopamine and serotonin in fatigue research. The poor correlation between LEDD and fatigue severity, coupled with the lack of significant serotonergic involvement, suggests that non‐dopaminergic systems—particularly noradrenaline and glutamate pathways—are likely to be more directly implicated in fatigue pathophysiology. This is further supported by the limited efficacy of serotonergic treatments, such as selective serotonin reuptake inhibitors, in alleviating PD‐related fatigue.^21^, ^22^

### 3.2 Implications for Fatigue Models

Previous studies have already demonstrated altered connectivity in the SMA and DMN of de novo PD fatigued patients^5^, ^6^ reinforcing the relevance of these networks in PD‐related fatigue. Our results further support these findings, highlighting that disruptions in these areas are central to the amplified perception of effort and fatigue, underscoring their role in the pathophysiology of PD fatigue. In this context, our insights expand the most recent model of fatigue, which suggests that disruptions in sensory attenuation during voluntary movements amplify the perception of effort.^10^ Noradrenergic dysfunction is closely linked with effort perception and may lead to fatigue by impairing PD patients' ability to efficiently mobilize resources for action.^55^ This dysfunction likely contributes to an increased perception of fatigue, as noradrenaline plays a crucial role in modulating attentional and arousal systems. Therefore, the role of noradrenaline in effort regulation offers a novel framework for understanding the pathophysiology of fatigue in PD.^10^ Our study further supports these findings, showing reduced mGluR5‐enriched connectivity in the SMA and widespread alterations in NET‐enriched connectivity in the SMN.

### 3.3 Limitations and Future Directions

Several limitations to this study should be acknowledged. First, the relatively small sample size and the lack of a control group may limit the generalizability of our findings. However, we verified that group differences were not driven by extreme values and applied nonparametric permutation tests to mitigate statistical biases.

Moreover, we excluded patients in more advanced stages of PD, which might have led to an incomplete representation of fatigue across the full disease spectrum.

Although levodopa is not directly associated with fatigue severity, its potential impact on functional connectivity, particularly within motor and associative networks, remains a methodological consideration. While we controlled for LEDD and included a mixed‐treatment patient group, the long‐term effects of dopaminergic therapy may still have influenced our results. Notably, levodopa also increases noradrenaline synthesis capacity, potentially affecting the role of the noradrenergic system in fatigue. By focusing on neurotransmitter‐enriched connectivity rather than whole‐brain FC patterns, we aimed to reduce the likelihood of global levodopa‐induced effects. However, as our study was conducted in medicated patients, we cannot fully disentangle medication effects. Future research should explore off‐medication conditions and longitudinal changes to better clarify the influence of dopaminergic treatment on fatigue‐related FC alterations.

However, our study offers key insights into the understanding of PD‐related fatigue. It is the first to establish a solid neurochemical basis for fatigue in PD, utilizing fatigue‐specific scales and controlling for confounding factors. Moreover, our findings provide a foundation for future therapies targeting the noradrenaline and glutamate systems. Drugs like atomoxetine, which has not yet been tested for PD‐related fatigue, and safinamide, which has already shown efficacy,^19^, ^20^ emerge as promising treatment options. The study also uniquely focuses on non‐de novo PD patients, broadening the applicability of our results to a wider spectrum of PD patients, including those on stable dopaminergic therapy.

Finally, our research confirms the involvement of the SMA in the sensorimotor network and sheds new light on the DMN, especially regions like the precuneus and posterior cingulate cortex, which are key in the perception of effort and fatigue. These areas could serve as a neurostimulation target in future works.

## 4 Conclusions

In conclusion, our study highlights the critical role of both mGluR5 and NET connectivity alterations in the SMA and other key regions of the DMN, salience, and sensorimotor networks in the development of PD‐related fatigue. These findings suggest a novel mechanism underlying the pathogenesis of fatigue in PD. Future research aimed at exploring the therapeutic potential of modulating mGluR5 and NET activity could pave the way for innovative, targeted treatments to manage fatigue more effectively in PD patients.

## Author Roles

(1) Research Project: A. Conceptualization, B. Methodology, C. Software, D. Data Curation; (2) Statistical Analysis: A. Design, B. Execution, C. Review and Critique; (3) Manuscript Preparation: A. Writing of the First Draft, B. Review and Critique.

I.A.D.V.: 1A, 1D, 2A, 3A, 3B.

M.M.: 1A, 1B, 1C, 1D, 2A, 2B, 2C, 3A, 3B.

A.T.: 1D, 3B.

G.T.: 1D, 3B.

G.B.: 1D, 3B.

D.M.: 1B, 2A, 3B.

O.D.: 1B, 1C, 2A, 3B.

M.V.: 1B, 3B.

A.B.: 1D, 3B.

E.M.: 1D, 3B.

A.S.: 3B.

S.O.: 1D, 3B.

F.B.P.: 1D, 3B.

M.T.: 1A, 1D, 3B.

M.C.: 1A, 1B, 1C, 1D, 2A, 2C, 3B.

## Financial Disclosures

None.

## Supporting information


**Data S1.** Supporting Information.

## Data Availability

The data that support the findings of this study are available from the corresponding author upon reasonable request. The REACT‐fMRI package is available at: https://github.com/ottaviadipasquale/react-fmri.
