# Supplementary material for: Molecular‐Informed Network Analysis Unveils Fatigue‐Related Functional Connectivity in Parkinson's Disease
Source: Mov Disord. 2025 Apr 22;40(8):1561–71. doi: 10.1002/mds.30214 (PMC12371637; doi:10.1002/mds.30214)
Supplement: Supplementary file 1 — Data S1. Supporting Information. [file MDS-40-1561-s001.docx]

**SUPPLEMENTARY MATERIAL**

**Molecular-informed network analysis unveils fatigue-related functional connectivity in Parkinson’s Disease**

I. A. Di Vico*; M. Moretto*; A. Tamanti; G. Tomelleri; G. Burati; D. Martins; O. Dipasquale; M. Veronese; A. Bertoldo; E. Menini; A. Sandri; S. Ottaviani; F. B. Pizzini; M. Tinazzi^+^; M. Castellaro^+^

**Manuela Moretto and Ilaria Antonella Di Vico contributed equally to this work.*

**Michele Tinazzi and Marco Castellaro contributed equally to this work.*

*Table 1: Overlay between significant clusters in the NET-enriched FC and mGluR5-enriched FC with Schaefer’s networks ……………………………………………...……2*

*Table 2: Overlay between significant clusters in the NET-enriched FC and mGluR5-enriched FC with AAL3’s regions …………….…………………………...………………2*

*Figure 1: Within group associations between NET-enriched and mGluR5-enriched FC and FSS, adding AES scores to the covariates………………………………………………………………………………..…...7*

*Figure 2: Within group associations between molecular-enriched FC and HDS / HAS…………………………………………………………………………………………...8*

| **Schaefer’s**  **functional network** | **% overlay with NET-enriched FC** | **% overlay with mGluR5-enriched FC** |
| --- | --- | --- |
| Control | 12.7 | 0.3 |
| DMN | 21.2 | 0.05 |
| Dorsal attention | 7.9 | 13.5 |
| Limbic | 0.02 | 0 |
| Salience | 28 | 23.5 |
| Somatomotor | 29.6 | 60.8 |
| Visual | 0.5 | 1.8 |
|  |  |  |

**Table 1. Percentage overlay between statistically significant areas in the NET-enriched FC (second column) and mGluR5-enriched FC (third column) and 7 cortical functional networks from Schaefer’s atlas.**

| **AAL3**  **anatomical region** | **% overlay with NET-enriched FC** | **% overlay with mGluR5-enriched FC** |
| --- | --- | --- |
| 'Precentral_L' | - | 2.90 |
| 'Precentral_R' | 0.85 | **11.19** |
| 'Frontal_Sup_2_L' | **5.18** | 1.65 |
| 'Frontal_Sup_2_R' | 0.04 | **6.84** |
| 'Frontal_Mid_2_L' | 3.18 | 0.15 |
| 'Rolandic_Oper_R' | 2.16 | - |
| 'Supp_Motor_Area_L' | 1.67 | **24.23** |
| 'Supp_Motor_Area_R' | 1.19 | **18.93** |
| 'Frontal_Sup_Medial_L' | 1.76 | - |
| 'Frontal_Sup_Medial_R' | 0.19 | - |
| 'Rectus_L' | 0.01 | - |
| 'Insula_R' | 0.74 | - |
| 'Cingulate_Mid_L' | **5.82** | 6.24 |
| 'Cingulate_Mid_R' | **4.75** | **8.64** |
| 'Cingulate_Post_L' | 1.12 | - |
| 'Cingulate_Post_R' | 0.58 | - |
| 'Hippocampus_R' | 0.01 | - |
| 'Cuneus_L' | 0.12 | 0.05 |
| 'Lingual_L' | 0.10 | - |
| 'Lingual_R' | 0.11 | - |
| 'Occipital_Sup_L' | - | 2.75 |
| 'Occipital_Mid_L' | - | 1.45 |
| 'Fusiform_L' | 0.05 | - |
| 'Postcentral_L' | 1.39 | 0.45 |
| 'Postcentral_R' | 1.51 | 3.00 |
| 'Parietal_Sup_L' | 1.27 | 1.35 |
| 'Parietal_Sup_R' | 0.01 | - |
| 'Parietal_Inf_L' | 0.11 | - |
| 'SupraMarginal_L' | 0.38 | - |
| 'SupraMarginal_R' | 2.19 | - |
| 'Angular_L' | 0.11 | - |
| 'Precuneus_L' | 4.80 | 3.75 |
| 'Precuneus_R' | 0.93 | - |
| 'Paracentral_Lobule_L' | 1.93 | 5.24 |
| 'Paracentral_Lobule_R' | 2.09 | 1.20 |
| 'Caudate_L' | 0.19 | - |
| 'Caudate_R' | 1.40 | - |
| 'Putamen_L' | 0.85 | - |
| 'Pallidum_L' | 0.03 | - |
| 'Pallidum_R' | 0.29 | - |
| 'Heschl_R' | 0.60 | - |
| 'Temporal_Sup_L' | 0.01 | - |
| 'Temporal_Sup_R' | 0.46 | - |
| 'Cerebelum_Crus1_L' | **5.06** | - |
| 'Cerebelum_Crus1_R' | 2.69 | - |
| 'Cerebelum_Crus2_L' | 2.57 | - |
| 'Cerebelum_Crus2_R' | 0.43 | - |
| 'Cerebelum_3_L' | 0.36 | - |
| 'Cerebelum_3_R' | 0.77 | - |
| 'Cerebelum_4_5_L' | 3.59 | - |
| 'Cerebelum_4_5_R' | 2.52 | - |
| 'Cerebelum_6_L' | 4.56 | - |
| 'Cerebelum_6_R' | 3.17 | - |
| 'Cerebelum_7b_L' | 0.80 | - |
| 'Cerebelum_7b_R' | 0.23 | - |
| 'Cerebelum_8_L' | 3.56 | - |
| 'Cerebelum_8_R' | **5.56** | - |
| 'Cerebelum_9_L' | 0.64 | - |
| 'Cerebelum_9_R' | 0.72 | - |
| 'Cerebelum_10_L' | 0.12 | - |
| 'Cerebellum_10_R' | 0.23 | - |
| 'Vermis_1_2' | 0.15 | - |
| 'Vermis_3' | 0.70 | - |
| 'Vermis_4_5' | 1.38 | - |
| 'Vermis_6' | 1.18 | - |
| 'Vermis_7' | 0.31 | - |
| 'Vermis_8' | 0.80 | - |
| 'Vermis_9' | 0.52 | - |
| 'Vermis_10' | 0.02 | - |
| 'Thal_AV_L' | 0.17 | - |
| 'Thal_AV_R' | 0.16 | - |
| 'Thal_LP_L' | 0.06 | - |
| 'Thal_LP_R' | 0.07 | - |
| 'Thal_VA_L' | 0.27 | - |
| 'Thal_VA_R' | 0.25 | - |
| 'Thal_VL_L' | 0.70 | - |
| 'Thal_VL_R' | 0.75 | - |
| 'Thal_VPL_L' | 0.10 | - |
| 'Thal_VPL_R' | 0.15 | - |
| 'Thal_IL_L' | 0.08 | - |
| 'Thal_IL_R' | 0.10 | - |
| 'Thal_MDm_L' | 0.29 | - |
| 'Thal_MDm_R' | 0.31 | - |
| 'Thal_MDl_L' | 0.08 | - |
| 'Thal_MDl_R' | 0.02 | - |
| 'Thal_MGN_R' | 0.02 | - |
| 'Thal_PuM_L' | 0.62 | - |
| 'Thal_PuM_R' | 0.27 | - |
| 'Thal_PuL_L' | 0.10 | - |
| 'Thal_PuL_R' | 0.01 | - |
| 'Thal_PuI_R' | 0.06 | - |
| 'ACC_pre_L' | 0.66 | - |
| 'ACC_pre_R' | 0.23 | - |
| 'ACC_sup_L' | 0.97 | - |
| 'ACC_sup_R' | 0.62 | - |
| 'Vent_Str_L' | 0.04 | - |
| 'SN_pr_L' | 0.02 | - |
| 'Red_N_L' | 0.01 | - |
| 'Red_N_R' | 0.01 | - |
| 'LC_R' | 0.01 | - |

**Table 2: Percentage overlap between statistically significant areas in the NET-enriched FC (second column) and mGluR5-enriched FC (third column) and anatomical ROIs from the AAL3 atlas.** Of the 170 ROIs, only those with an overlap percentage greater than 0 are displayed. The five highest values are highlighted in bold.

**Within group associations between molecular-enriched FC and FSS, adding AES scores to the covariates**

We repeated the within-group statistical test to look for association between FSS and molecular-enriched FC, adding the apathy scores (AES) to the covariates of the main model (age, sex, LEDD, and eICV). For dopamine and serotonin, we did not find any significant relationship between FSS and connectivity.

Whereas, for NET- and mGluR5-enriched FC we found significant negative relationship between FSS scores and connectivity, in regions overlapping the ones found with the main model (see Figure 1 of Manuscript).

For NET, we found significant relationships in the left and right middle cingulate cortex/gyrus, right cerebellum, left superior frontal cortex/gyrus, left precuneus (see Figure 1, top panel). When assessing the overlay between statistically significant voxels and cortical functional networks, as defined in the Schaefer functional atlas, we found a major involvement of areas belonging to the Somatomotor (45.07% overlay), Salience (22.85% overlay), and Default Mode Network (13.09% overlay) networks.

While, for mGluR5, we found significant relationships in the left and right supplementary motor area, right and left precentral and postcentral cortex, left and right paracentral lobule, middle cingulate cortex (see Figure 1, bottom panel). When assessing the overlay between statistically significant voxels and cortical functional networks, as defined in the Schaefer functional atlas, we found a major involvement of areas belonging to the Somatomotor (62.68% overlay), Salience (31.29% overlay), and Dorsal Attention (5.33% overlay) networks.

**
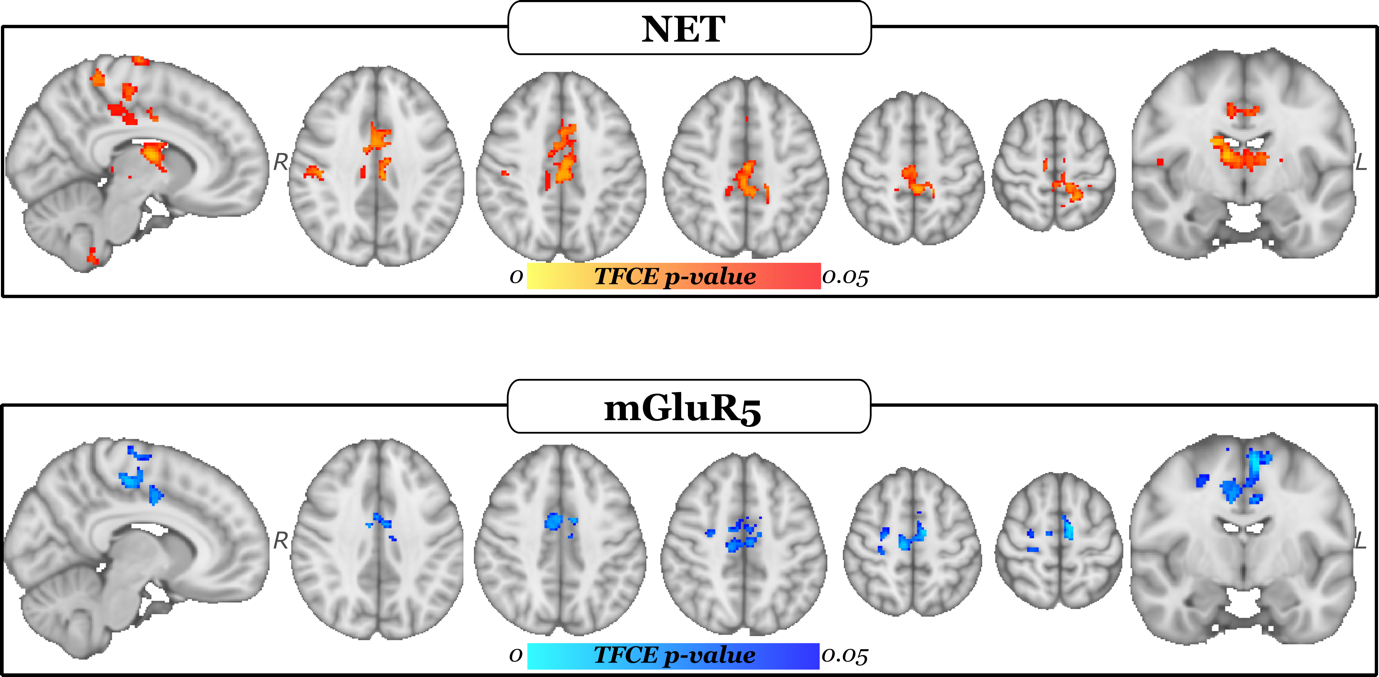
**

**Figure 1. Decrease NET-enriched and mGluR5-FC with increasing fatigue.** Clusters of statistically significant decrease of NET-enriched FC (top row) and mGluR5 (bottom row) with increasing fatigue. The color scales represent p-values after correction for multiple comparisons. In the top row, warmer colors indicate more significant voxels. Similarly, in the bottom row, lighter colors indicate more significant voxels. The statistical maps are overlaid onto the MNI atlas.

**Within group associations between molecular-enriched FC and HDS / HAS**

Since the depression (HDS) scale correlated with the fatigue severity scale (FSS), and thus HDS cannot be included as regressors in the statistical models to avoid removing variance, we repeated the statistical tests using HDS as explanatory variable instead of FSS, using age, sex, LEDD, and eICV as covariates. For the NET-enriched FC we did not find any significant relationship between HDS scores and connectivity. While, for mGluR5, we found significant relationships in the left and right supplementary motor area, left precentral and postcentral cortex, left and right paracentral lobule, middle cingulate cortex (see Figure 2). When assessing the overlay between statistically significant voxels and cortical functional networks, as defined in the Schaefer functional atlas, we found a major involvement of areas belonging to the Somatomotor (64.49% overlay), Salience (17.55% overlay), and Dorsal Attention (17.38% overlay) networks.


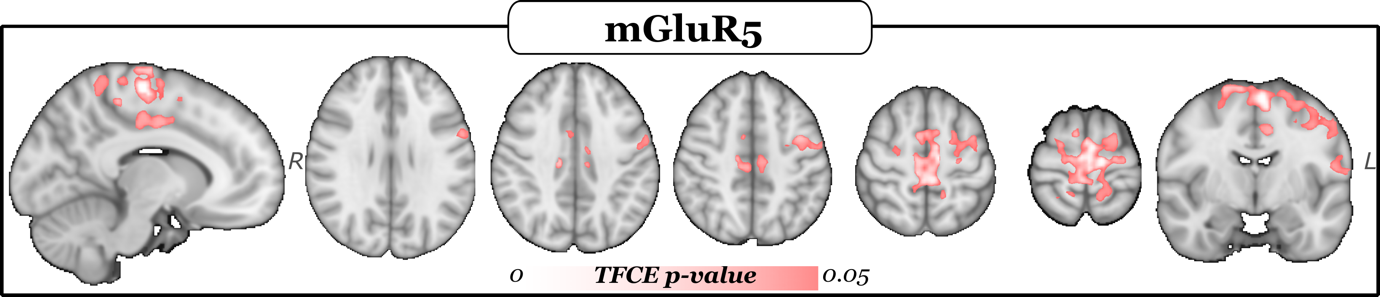


**Figure 2. Decrease mGluR5-enriched FC with increasing depression.** Clusters of statistically significant decrease of mGluR5 with increasing HDS scores. The color scales represent p-values after correction for multiple comparisons. In the top row, warmer colors indicate more significant voxels. Similarly, in the bottom row, lighter colors indicate more significant voxels. The statistical maps are overlaid onto the MNI atlas.
